# Supplementary material for: Gating of TonB-dependent transporters by substrate-specific forced remodelling
Source: Nat Commun. 2017 Apr 21;8:14804. doi: 10.1038/ncomms14804 (PMC5413942; doi:10.1038/ncomms14804)
Supplement: Supplementary Information — Supplementary Figures, Supplementary Tables. [file ncomms14804-s1.pdf]

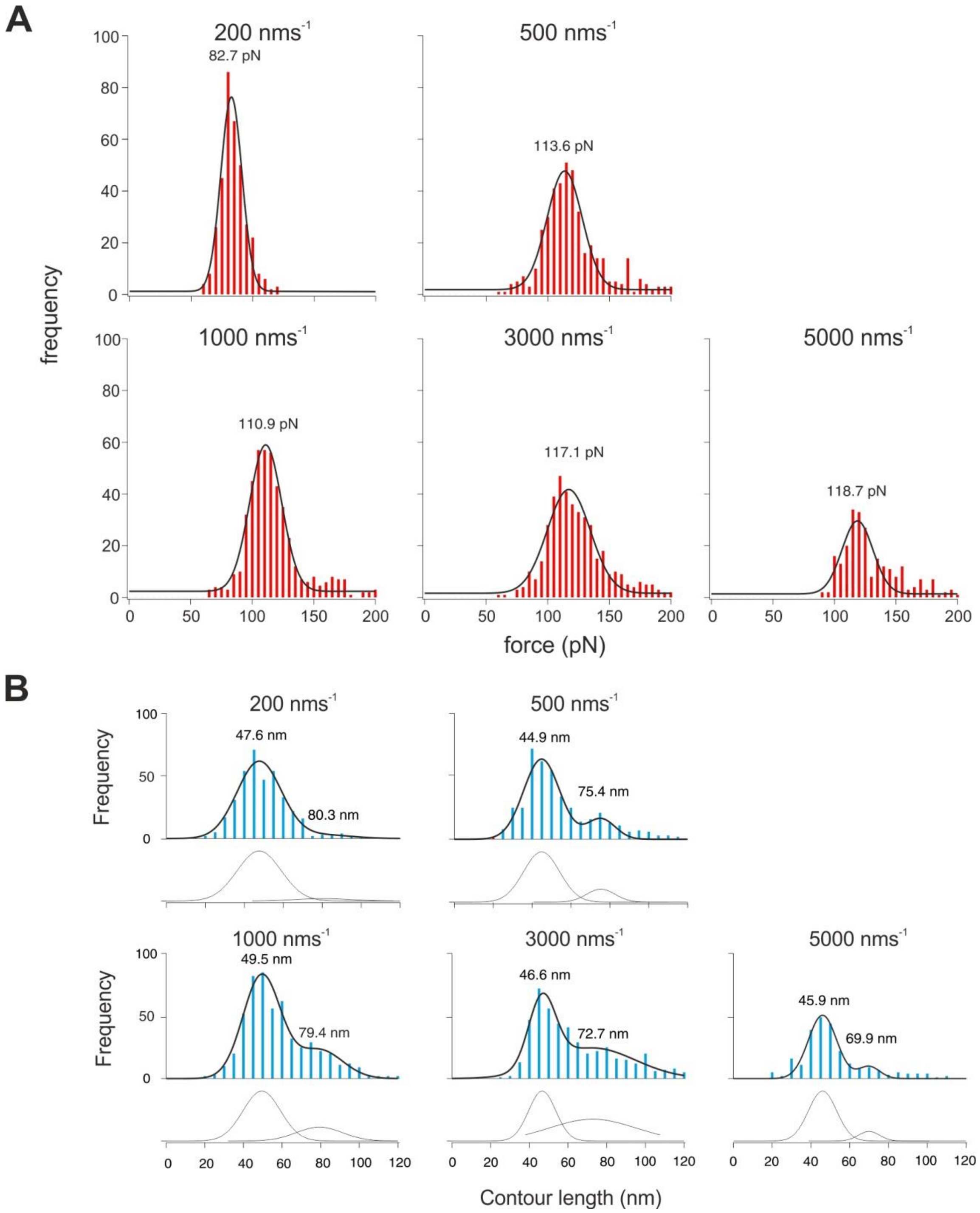

Supplementary Fig. 1: A) Force-frequency distributions (red bars) for TB_{BtuB}:$TonB_{\Delta TMD}$ dissociation events with fits to a single Gaussian distribution for the five pulling velocities (200, 500, 1000, 3000, and 5000 nms^{-1}) used in this study. **B)** Contour-frequency distributions (blue bars) for TB_{BtuB}:$TonB_{\Delta TMD}$ dissociation events fitted to multiple Gaussian distributions for the five pulling velocities (200, 500, 1000, 3000, and 5000 nms^{-1}) used in this study. The deconvoluted Gaussians are shown below. Histograms contain the full triplicate data collected.

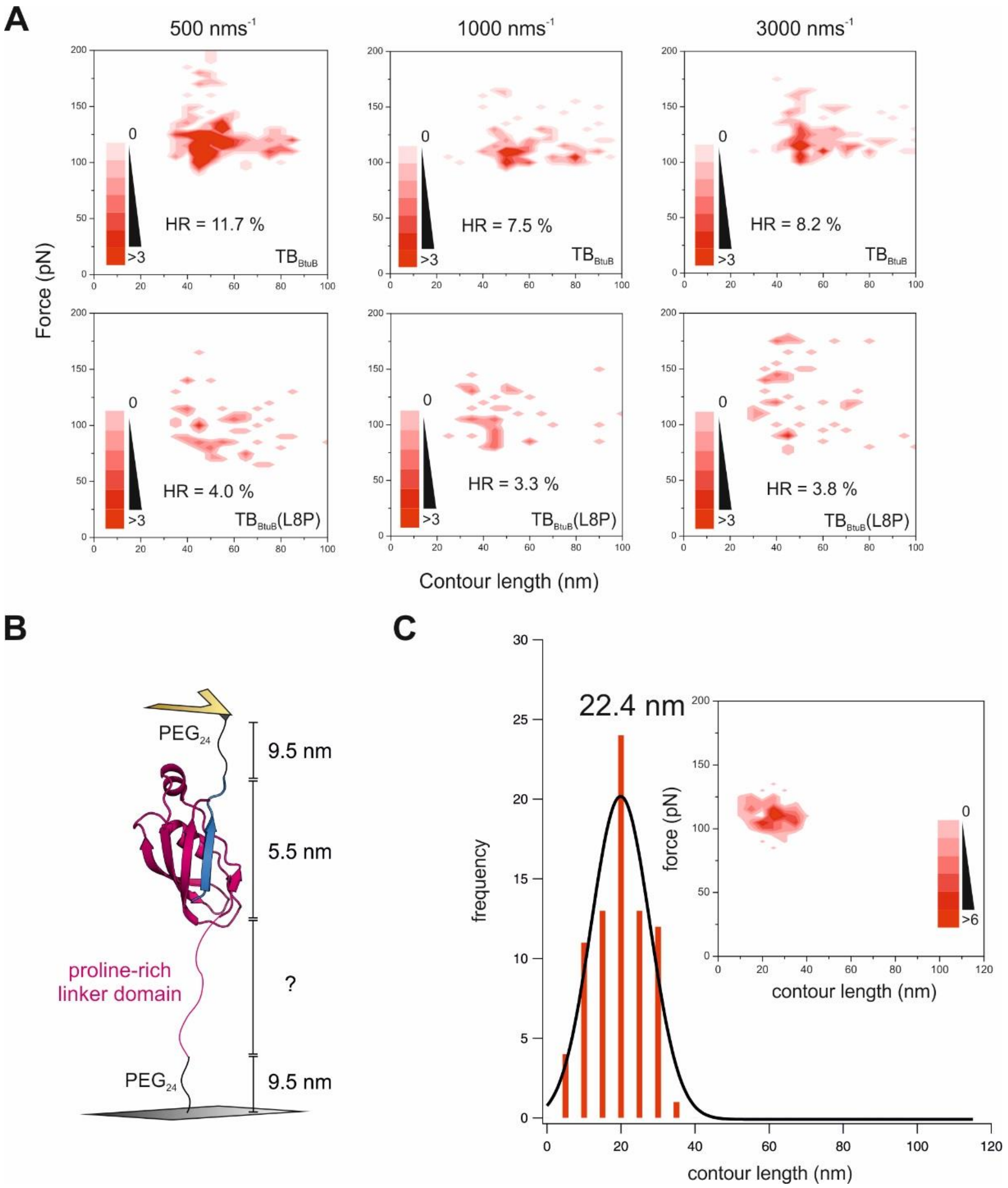

Supplementary Fig. 2: A) Force spectroscopy data (rupture force and contour length) of TB_{BtuB} (top) and L8P TB_{BtuB} (bottom) unbinding from $TonB_{\Delta TMD}$ plot as 2D contour maps at three different retraction velocities. Each contour map contains all filtered data reflecting single-molecule dissociation events after 1200 surface-approaches over a large surface area. The 'hot spot' of specific unbinding events is lost in the L8P variant. The hit rate (HR) is shown in the inset of each graph. The points were binned by a contour length of 5 nm and force of 10 pN, the number of points that occupy a quadrant are indicated by the colour of the contours. **B)** Individual lengths of each component. **C)** To verify that the bimodal contour length distributions evident in Supplementary Figs. 1B and 2A were due to the presence of an extended and compact form of the linker region of TonB, a construct was created which only comprised the structured C-terminal domain of TonB (residues 145-239) (designated $TonB_{CTD}$). AFM was performed using the same conditions as the $TonB_{\Delta TMD}$ experiment and the resultant contour length-frequency distribution for TB_{BtuB}:$TonB_{CTD}$ dissociation events at a pulling velocity of 500 nms^{-1} fitted well to a single Gaussian. The inset shows a contour map of contour length vs rupture force for the same data set. Points were binned by a contour length of 5 nm and force of 10 pN and the number of points that occupy a quadrant are indicated by the colour of the contours. These data suggest that the linker region of TonB populates at least two mechanically distinct conformations (manuscript in preparation).

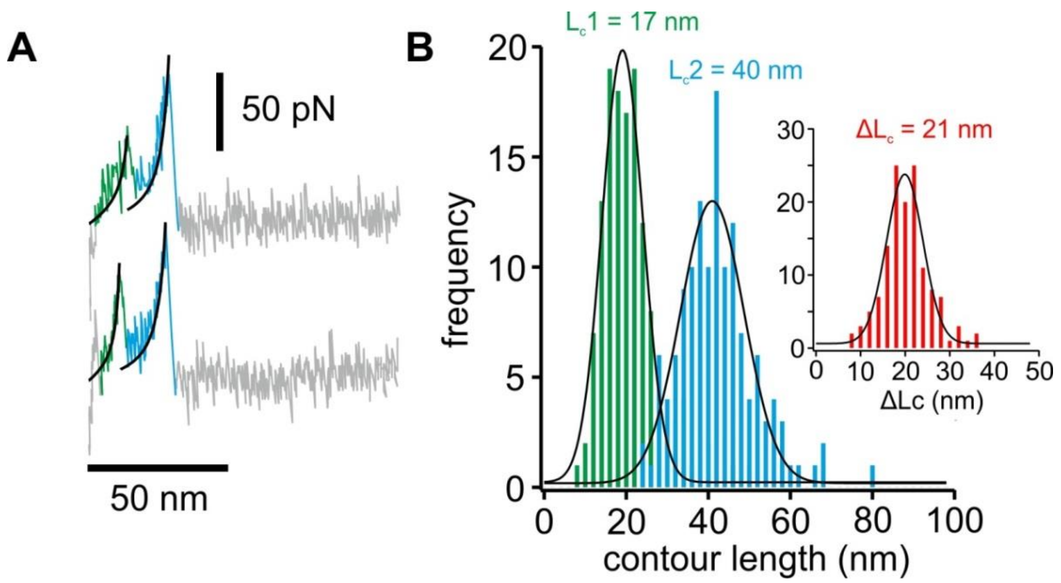

Supplementary Fig. 3: SMFS results of wild-type BtuB unfolding using $TonB_{CTD}$ construct at 1000 nms^{-1}. **A)** Example force-extension traces of $TonB_{CTD}$:BtuB:vitamin B_{12} with the worm-like chain model (p= 0.4 nm) fit. **B)** Contour length distributions of unfolding (green) and unbinding (blue) and ΔL_c (red inset).

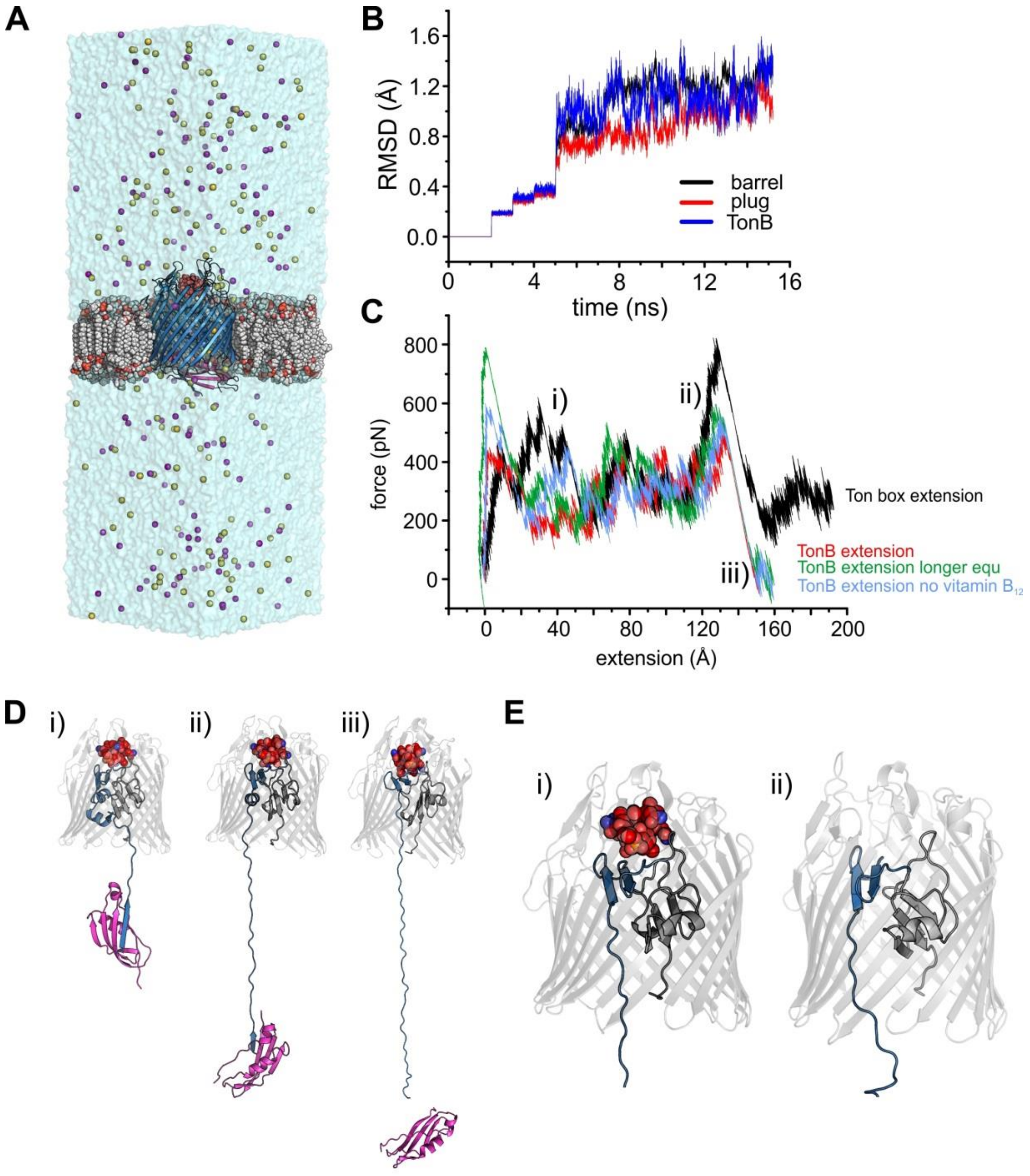

Supplementary Fig. 4: Steered molecular dynamics (SMD) of the TonB:BtuB:vitamin B_{12} complex. **A)** The equilibrated system of BtuB (blue cartoon) in complex with TonB (pink cartoon) and vitamin B_{12} (red space-fill) (PDB: 2GSK) in a POPC lipid bilayer (red and white spheres) and a large water box (blue surface representation). The Na^{+} and Ca^{2+} ions are shown as coloured spheres. **B)** Root mean square deviation (RMSD) of the individual components (labelled) during the equilibration of the system. **C)** SMD force-extension plot when extended via the Ton box (black), or from TonB (red). Extension via the Ton box caused sequential unfolding of the plug domain after 80 ns of simulation (corresponding to a 20 nm extension of the C-terminus from its original position) and formation of a channel through the receptor (Figure 5B). When extended from the centre of mass of TonB, it remained in complex with the Ton box and 46 residues (up to residue G51, ~13 nm of extension) of the plug domain was unfolded before the complex broke apart. To form a continuous channel, an additional 22 residues are required to be unfolded (up to residue S74). A longer equilibration (20 ns) of the system prior to extension (green force extension profile), however, did not change this observation. Finally to assess whether vitamin B_{12} was stabilising residues 47-74 (which it is also in contact with) a system was set up without the vitamin B_{12} in the BtuB binding pocket (blue force extension profile). Again, 46 residues were unfolded before TonB dissociated from the Ton box. **D)** SMD snap-shots of TonB (pink) unfolding the plug domain of BtuB (barrel domain shown as grey transparent cartoon, plug domain shown in blue and vitamin B_{12} as spheres. The membrane and water are hidden for simplicity). In iii) 46 residues (~130 Å extension) have been unfolded by the non-covalent TonB:Ton box complex. The numbered snapshots are also shown in C)**. E)** Close up of the BtuB plug domain i) with vitamin B_{12} and ii) without) after 130 Å extension via SMD using TonB, BtuB plug residues 5-74 are shown in blue and the recalcitrant plug subdomain in grey.

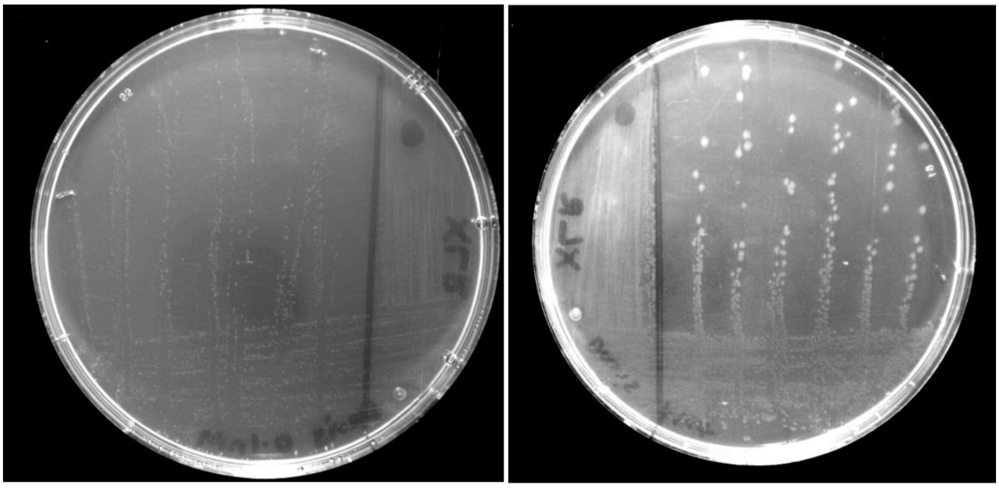

Davis 0.1 nM vitamin B_{12} Davis 5 µg/mL methionine

\+ ++

Supplementary Fig. 5: Examples of the methionine growth assay on Davis minimal agar with 0.1 nM vitamin B_{12} after 48 hours incubation at 37 °C. **Left**: partial growth seen in a BtuB variant where partial plug domain unfolding is prevented. **Right**: the full growth phenotype (colony size 2 mm) is exemplified by the colonies observed after incubated for 48 hours at 37 °C on methionine supplemented Davis minimal agar (right).

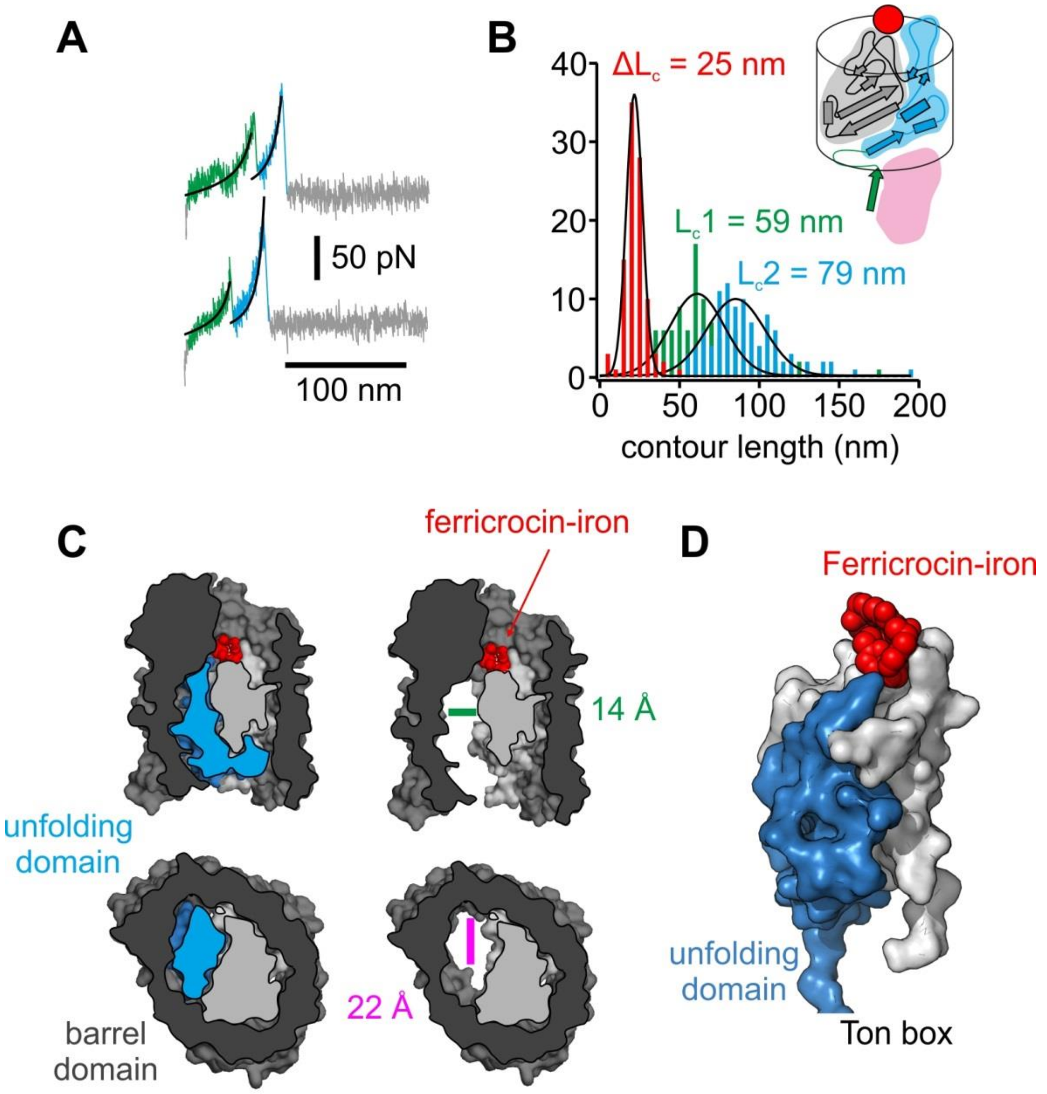

Supplementary Fig. 6: SMFS of FhuA **A)** Examples of the double rupture events with the WLC fit **B)** L_c1 (green), L_c2 (blue) and ΔL_c (red) distributions of the double rupture peaks with Gaussian fits (data shown are from one replicate); the inset shows a secondary structure schemetic of the FhuA plug domain with the residues unfolded (inferred from the ΔL_c value) shown in blue, TonB in pink, the Ton box in green and the ferricrocin substrate in red. **C)** Surface representation vertical and horizontal cross-sections of FhuA (PDB: 2GRX) showing the effect of removing 62 amino acids (blue) downstream of the Ton box. The dimensions of the resultant channel are shown. **D)** Surface representation of the plug domain of FhuA, the residues that are unfolded (inferred from the ΔL_c value) are coloured blue.

					Plug unfolding				TonB unbinding					
BtuB wt	#	N attempts	n events*	Hits (%)	Force (pN)	Average	L_c (nm)	Average	Force (pN)	Average	L_c (nm)	Average	ΔL_c (nm)	Average
200 nms^{-1}	1	2400	33	1.4	62	51 ± 10	54	53 ± 4	68	66 ± 3	80	76 ± 7	21	19 ± 2
	2	4300	42	1.0	41		55		63		79		19	
	3	2237	19	0.8	51		49		66		69		18	
500 nms^{-1}	1	1200	30	2.5	70	59 ± 10	51	53± 6	77	85 ± 8	80	76 ± 9	26	22 ± 4
	2	1050	27	2.6	57		48		90		67		20	
	3	1167	24	2.0	49		59		87		81		20	
1000 nms^{-1}	1	2400	54	2.2	63	61 ± 4	58	58 ± 3	105	91 ± 23	70	77 ± 7	20	20 ± 2
	2	1200	18	1.5	62		55		68		80		21	
	3	1948	81	4.1	57		61		100		82		18	
5000 nms^{-1}	1	1000	17	1.7	130	118 ± 20	41	45 ± 5	125	131 ± 21	75	71 ± 10	24	24 ± 2
	2	1200	11	0.9	98		43		115		61		25	
	3	1200	44	3.7	125		50		152		76		22	

Supplementary Table 1:AFM data of TonB$_{\Delta TMD}$:wild-type BtuB unbinding at various retraction velocities.
Force, contour length (L_c) and ΔL_c values were calculated by plotting the distribution of events and fitting with a Gaussian to obtain the mean value. The error on the average is the range of the triplicate averages. *number of double events.

					Plug unfolding				TonB unbinding					
Variant	#	N attempts	n events[a]	Hits (%)	Force (pN)	Av	L_c (nm)	Average	Force (pN)	Average	L_c (nm)	Av	ΔL_c (nm)	Av
BtuB:TonB$_{CTD}$	1	9815	69	0.7	59	52 ±7	17	19 ± 2	94	86 ± 8	40	41 ± 2	21	20 ± 2
	2	4617	42	0.9	50		21		82		43		21	
	3	8550	26	0.3	48		20		82		40		18	

Supplementary Table 2: AFM data of TonB$_{CTD}$:wild-type BtuB at 1000 nms^{-1}.
Force, contour length (L_c) and ΔL_c values were calculated by plotting the distribution of events and fitting with a Gaussian to obtain the mean value. The error on the average is the range of the triplicate averages.

					Plug unfolding				TonB unbinding					
Variant	#	N attempts	n events[a]	Hits (%)	Force (pN)	Av	L_c (nm)	Average	Force (pN)	Average	L_c (nm)	Av	ΔL_c (nm)	Av
BtuB wt	1	2400	54	2.2	63	60 ± 3	58	57 ± 4	105	91 ± 23	70	77± 7	20	20 ± 2
	2	1200	18	1.5	62		54		68		80		21	
	3	1948	81	4.1	57		61		100		82		18	
XL_{barrel} (L23C/S374C) No DTT	1	6725	85[b]	1.3[b]	-	-	-	-	102	100 ± 6	48	47 ± 2	-	-
	2	2492	38[b]	1.5[b]	-		-		104		45		-	
	3	3304	73[b]	2.2[b]	-		-		94		47		-	
XL_{barrel} (L23C/S374C) 2 mM DTT	1	3419	18	0.5	63	64 ± 1	52	51 ± 5	92	99 ± 7	82	76 ± 7	25	21 ± 4
	2	3669	69	1.9	65		46		105		69		21	
	3	3753	22	0.6	64		55		100		77		19	
XL_{loop} (V29C/V45) No DTT	1	6386	88	1.4	56	55 ± 4	58	53 ± 7	103	98 ± 8	76	69 ± 8	17	14 ± 3
	2	7365	44	0.6	51		55		90		71		11	
	3	2000	22	1.1	58		46		101		61		15	
XL_{loop} (V29C/V45) 2 mM DTT	1	8324	34	0.4	60	61 ± 4	49	49 ± 3	99	102 ± 3	70	69 ± 4	21	20 ± 1
	2	6420	71	1.1	65		52		102		72		19	
	3	3579	17	0.5	57		47		105		65		19	

Supplementary Table 3: data of $TonB_{\Delta TMD}$:BtuB variants (± 2 mM DTT) at 1000 nms^{-1}.
Force, contour length (L_c) and ΔL_c values were calculated by plotting the distribution of events and fitting with a Gaussian to obtain the mean value. The error on the average is the range of the triplicate averages. [a]number of double events, [b]number/% of single events.

					Plug unfolding				TonB unbinding					
Protein	#	N attempts	n events[a]	Hits (%)	Force (pN)	Av	L_c (nm)	Average	Force (pN)	Average	L_c (nm)	Av	ΔL_c (nm)	Av
FhuA	1	5207	34	0.7	89	74 ± 11	66	62 ± 4	124	105 ± 24	94	85 ± 9	26	25 ± 1
	2	6139	41	0.7	70		62		100		82		24	
	3	8150	24	0.3	63		59		91		79		24	

Supplementary Table 4: AFM data of $TonB_{\Delta TMD}$:FhuA at 1000 nms^{-1}.
Force, contour length (L_c) and ΔL_c values were calculated by plotting the distribution of events and fitting with a Gaussian to obtain the mean value. The error on the average is the range of the triplicate averages.

		Peak 1		Peak 2		Peak 3 (TonB unbinding)			
protein	#	Force (pN)	L_c (nm)	Force (pN)	L_c (nm)	Force (pN)	L_c (nm)	ΔL_c 1 (nm)	ΔL_c 2 (nm)
BtuB I80A/L85A/L96A	1	68	50	85	75	117	98	23	28
	2	62	51	96	72	120	94	17	23
	3	62	49	99	62	125	90	18	27
	av	64 ± 4	50 ± 1	94 ± 9	70 ± 5	121 ± 4	94 ± 4	19 ± 4	26 ± 3

Supplementary Table 5: AFM data of $TonB_{\Delta TMD}$:$BtuB_{3A}$ at 1000 nms^{-1}.
Force, contour length (L_c) and ΔL_c values were calculated by plotting the distribution of events and fitting with a Gaussian to obtain the mean value. The error on the average is the range of the triplicate averages.

Variant	Forward primer	Reverse primer
BtuB L8P	CCCGGATACTCCCGTCGTTACTG	CTGGTATCCTGTGCCCAA
BtuB L23C	CAGCACTGTGTGTGCACCAACCACC	CGCGGCTGTTCAAAACGG
BtuB S374C	CTGGCAAACCTGCGCCGGTTG	GTTCCATGACGACCAAACTGTG
BtuB V29C	AACCACCGTTTGCACCCGTCAGGATATCGAC	GGTGCAAGCACAGTGCTG
BtuB V45C	GGTCAATGATTGCCTGCGCCGTCTTCCGGG	GAGGTCGACTGCCAGCGG
BtuB I80A/L85A	GTACGCGCGAATCTGGCGGGGGTGAGT	GCCATCAGCTAACACCAACACATGACTGGC
BtuB L96A	TTCTGCCGACGCTAGCCAGTTCCCTATTGCG	CCACTCACCCCCGCCAGA

Supplementary Table 6: Primers for generating BtuB variants using Q5® Site-Directed Mutagenesis kit (NEB)
